# Supplementary material for: The Effect of Imidacloprid on the Volatile Organic Compound Profile of Strawberries: New Insights from Flavoromics
Source: Foods. 2023 Jul 31;12(15):2914. doi: 10.3390/foods12152914 (PMC10418971; doi:10.3390/foods12152914)
Supplement: Supplementary file 1 [file foods-12-02914-s001.zip › 02-Supporting information-Figure S1-S3.pdf]

Supporting Information

New insights from flavoromics on strawberries: different volatile organic compounds profile under organic and conventional agricultural practices

Ning Yue[a, #], Hongping Wang[a, #], Chunmei Li[b], Chen Zhang[a], Simeng Li[a], Jing Wang[a], Fen Jin[a, *]

[a] Key Laboratory of Agro-product Quality and Safety, Institute of Quality Standards & Testing Technology for Agro-products, Chinese Academy of Agricultural Sciences, Beijing 100081, China

[b] Institute of Food Science and Technology, Chinese Academy of Agricultural Sciences, Beijing 100193, China

* Corresponding author:

Fen Jin

E-mail: jinfenbj@163.com; Tel: +86-10-8210-6502

Figure S1 The calibration curve of external standard with GC×GC-TOFMS by headspace SPME

Figure S2 Chromatograms and mass spectra of esters (Allyl Isothiocyanate and Ethyl butyrate), ketones (3-Nonen-2-one), aldehydes (Decanal), terpenes (α-Terpineol), and furanones (DMMF)

Figure S3 PCA and PLS-DA analysis in IMI treated and non-IMI treated strawberries obtained with GC × GC-TOFMS.

Materials and chemicals

The 20-mL headspace vials covered with 18 mm magnetic PTFE/silicone caps were supplied by Ingenious Laboratory Technology Co. Ltd. (Guangzhou, China). Imidacloprid (IMI) was purchased from Tide Crop Science Co. Ltd. (Zhejiang, China). Distilled water was purchased from A. S. Watson Group Ltd. (Guangzhou, China). Straight-chain alkanes (C7–C40) purchased from Sigma-Aldrich (Missouri, USA) were employed for the determination of linear retention indices (LRIs). n-Hexane was purchased from Thermo Fisher Scientific (Massachusetts, USA). According to the classification of substances, the key aroma compounds were selected and adopted for external standard calibration, including 2,5-Dimethyl-4-methoxy-3(2H)-furanone (DMMF), benzaldehyde, 2-heptanone, 1-decene, 1-heptanol, eugenol, ethyl hexanoate, and 1-teradecanol standard, which were purchased from TCI Lot. (>97%, Shanghai, China).

Headspace solid-phase microextraction procedure

The VOCs were detected by GC×GC TOFMS (GGT 0620) (Hexin Analytical Instrument Co., Ltd., Guangzhou, China), which was equipped with a GERSTEL MPS 2XL auto-sampler device (Ingenious Laboratory Technology Co. Ltd., Guangzhou, China). The extraction method was carried out on the basis of previous report, homogenized strawberry fruit sample (2.0 g) was placed into a 20 mL headspace vial sealed with screw caps. After equilibration at 55 °C for 20 min, the SPME fiber was immediately inserted into the injector port at 250 °C for 5 min after extraction for 30 min at 55 °C (WR/PDMS, 50/30 μm, 10 mm; Ingenious Laboratory Technology Co. Ltd., Guangzhou, China).

Data processed:

Canvas Browser version W2.2.0.29702 software (Xuejing Electronic Technology Co., Ltd., Shanghai, China); Automated peak finding and spectral deconvolution:3.71; Signal-to-noise ratio: 7; The maximum one- and two-dimension retention time deviations: 12 s and 0.2 s, respectively. LRIs: n-alkanes (C7-C40); LRI values in the error range: 30 (NIST library); Database: NIST 20 databases; Minimum match: 700; The peak area ratios of any volatiles quantified the relative contents of volatile compounds.

All the experimental samples were run in duplicate. The principal component analysis (PCA) and partial least squares discriminant analysis (PLS-DA) were carried out with Hexin Mass Data Trace software (Hexin Analytical Instrument Co., Ltd., Guangzhou, China). One-way analysis of variance (ANOVA) was used to analysis the significance between groups by SPSS Statistics 21.0 software (SPSS Inc., Chicago, IL, USA).

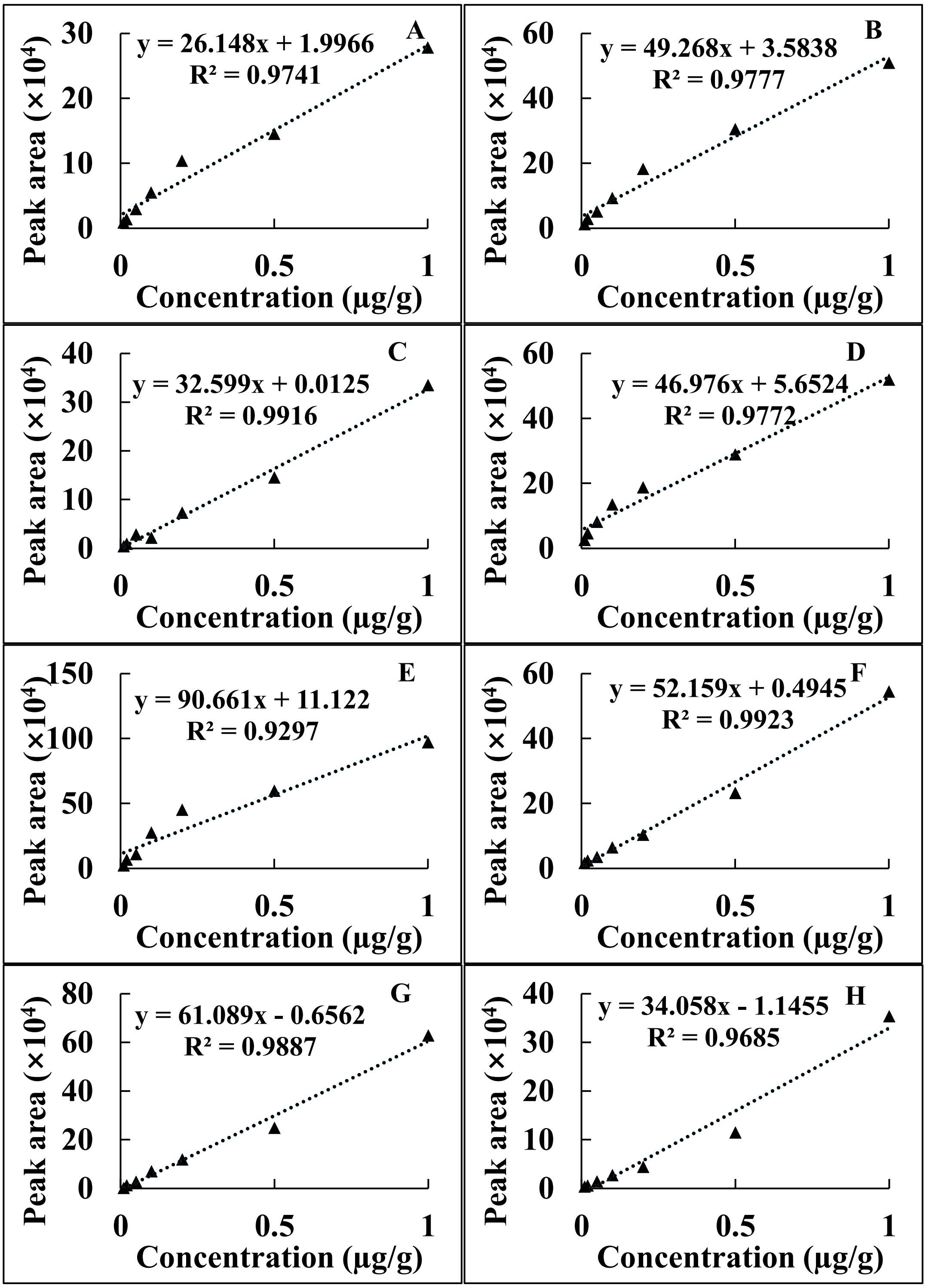

Figure S1 The calibration curve of external standard with GC×GC-TOFMS by headspace SPME

(A) 2-Heptanone; (B) Benzaldehyde; (C) 1-Heptanol; (D) 1-Decene; (E) Ethyl Hexanoate; (F) DMMF; (G) Eugenol; (H) 1-Teradecanol

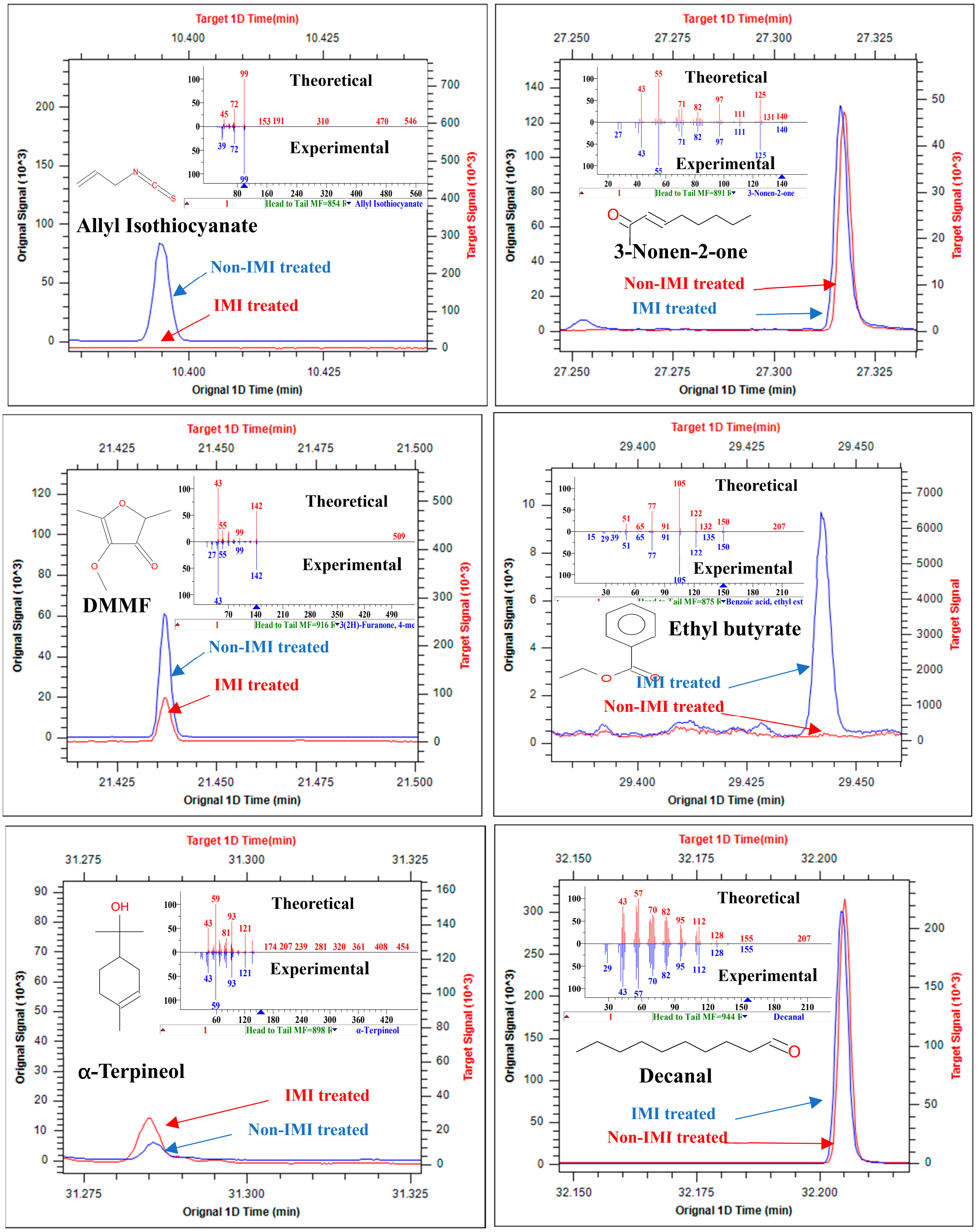

Figure S2 Chromatograms and mass spectra of esters (Allyl Isothiocyanate and Ethyl butyrate), ketones (3-Nonen-2-one), aldehydes (Decanal), terpenes (α-Terpineol), and furanones (DMMF).

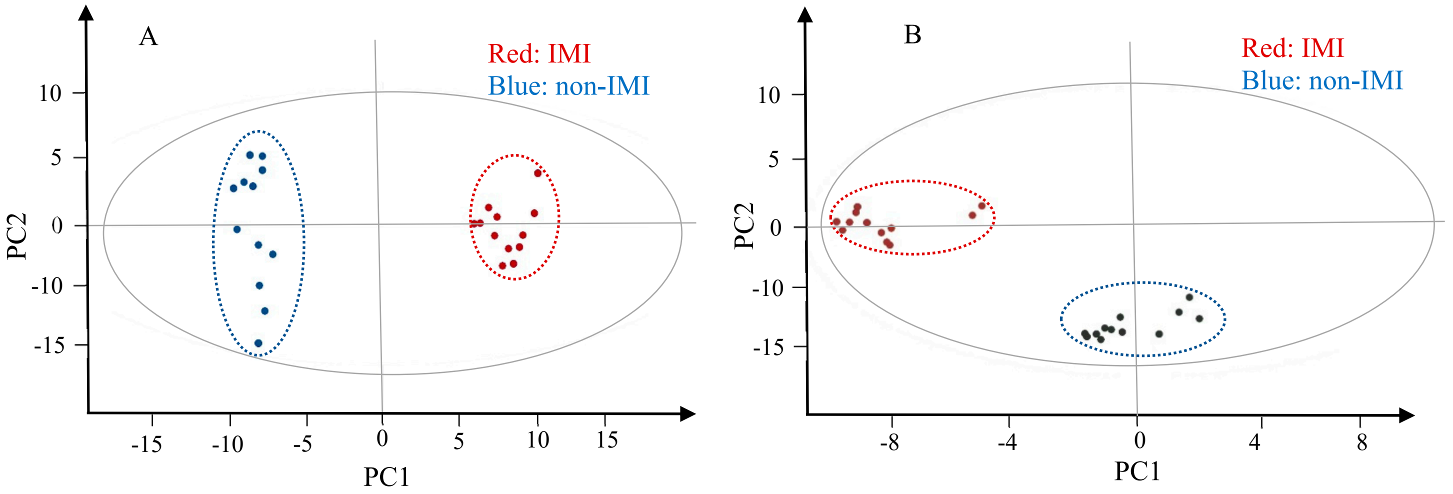

Figure S3 PCA and PLS-DA analysis in IMI treated and non-IMI treated strawberries obtained with GC × GC-TOFMS.
(A) score plots of PCA; (B) score plots of PLS-DA
