# Supplementary material for: The Effect of Imidacloprid on the Volatile Organic Compound Profile of Strawberries: New Insights from Flavoromics
Source: Foods. 2023 Jul 31;12(15):2914. doi: 10.3390/foods12152914 (PMC10418971; doi:10.3390/foods12152914)
Supplement: Supplementary file 1 [file foods-12-02914-s001.zip › 03-supporting information-Table S1.pdf]

Supporting Information

New insights from flavoromics on strawberries: different volatile organic compounds profile under organic and conventional agricultural practices

Ning Yue[a, #], Hongping Wang[a, #], Chunmei Li[b], Chen Zhang[a], Simeng Li[a], Jing Wang[a], Fen Jin[a, *]

[a] Key Laboratory of Agro-product Quality and Safety, Institute of Quality Standards & Testing Technology for Agro-products, Chinese Academy of Agricultural Sciences, Beijing 100081, China

[b] Institute of Food Science and Technology, Chinese Academy of Agricultural Sciences, Beijing 100193, China

* Corresponding author:

Fen Jin

E-mail: jinfenbj@163.com; Tel: +86-10-8210-6502

Table S1 The absolute recovery of external standard with GC×GC-TOFMS in the strawberry sample homogenates by headspace SPME

**Compounds**	**Concentration (μg/g)**	**Average absolute recovery (%)**	**RSD (%)**
2-Heptanone	0.5	91.89	4.77
	0.2	97.12	9.09
	0.1	132.48	4.43
Benzaldehyde	0.5	64.76	4.74
	0.2	84.08	16.87
	0.1	101.73	15.98
1-Heptanol	0.5	70.54	6.96
	0.2	85.03	8.99
	0.1	103.45	4.97
1-Decene	0.5	72.71	14.38
	0.2	108.57	5.00
	0.1	130.47	9.93
Ethyl Hexanoate	0.5	79.49	2.88
	0.2	126.98	0.48
	0.1	85.04	1.25
DMMF	0.5	82.73	3.52
	0.2	110.27	12.25
	0.1	89.82	6.79
Eugenol	0.5	69.09	11.57
	0.2	74.17	21.17
	0.1	83.75	14.35
1-Teradecanol	0.5	69.64	10.49
	0.2	79.36	7.93
	0.1	96.03	5.83
